# Experimental verification and comprehensive analysis of m7G methylation regulators in the subcluster classification of ischemic stroke

**DOI:** 10.3389/fgene.2022.1036345

**Published:** 2023-01-04

**Authors:** Yunze Tian, Beibei Yu, Boqiang Lv, Yongfeng Zhang, Longhui Fu, Shijie Yang, Jianzhong Li, Shouping Gong

**Affiliations:** ^1^ Department of Neurosurgery, The Second Affiliated Hospital of Xi’an Jiao Tong University, Xi’an, China; ^2^ Department of Thoracic Surgery, The Second Affiliated Hospital of Xi’an Jiao Tong University, Xi’an, China

**Keywords:** ischemic stroke, modification of 7-methylguanosine, immunity, consensus clustering, transcription factor

## Abstract

**Background:** Ischemic stroke (IS) is a fatal cerebrovascular disease involving several pathological mechanisms. Modification of 7-methylguanosine (m7G) has multiple regulatory functions. However, the expression pattern and mechanism of m7G in IS remain unknown. Herein, we aimed to explore the effect of m7G modification on IS.

**Methods:** We screened significantly different m7G-regulated genes in Gene Expression Omnibus datasets, GSE58294 and GSE22255. The random forest (RF) algorithm was selected to identify key m7G-regulated genes that were subsequently validated using the middle cerebral artery occlusion (MCAO) model and quantitative polymerase chain reaction (qPCR). A risk model was subsequently generated using key m7G-regulated genes. Then, “ConsensusClusterPlus” package was used to distinguish different m7G clusters of patients with IS. Simultaneously, between two m7G clusters, differentially expressed genes (DEGs) and immune infiltration differences were also explored. Finally, we investigated functional enrichment and the mRNA–miRNA–transcription factor network of DEGs.

**Results:** RF and qPCR confirmed that *EIF3D, CYFIP2, NCBP2, DCPS,* and *NUDT1* were key m7G-related genes in IS that could accurately predict clinical risk (area under the curve = 0.967). *NCBP2* was the most significantly associated gene with immune infiltration. Based on the expression profiles of these key m7G-related genes, the IS group could be divided into two clusters. According to the single-sample gene set enrichment analysis algorithm, four types of immune cells (immature dendritic cells, macrophages, natural killer T cells, and TH1 cells) were significantly different in the two m7G clusters. The functional enrichment of 282 DEGs between the two clusters was mainly concentrated in the “regulation of apoptotic signaling pathway,” “cellular response to DNA damage stimulus,” “adaptive immune system,” and “pyroptosis.” The miR-214*–LTF*–*FOXJ1* axis may be a key regulatory pathway for IS.

**Conclusion:** Our findings suggest that *EIF3D, CYFIP2, NCBP2, DCPS,* and *NUDT1* may serve as potential diagnostic biomarkers for IS and that the m7G clusters developed by these genes provide more evidence for the regulation of m7G in IS.

## 1 Introduction

Ischemic stroke (IS) is the most common cerebrovascular disease, with high mortality and morbidity. It affects approximately 15 million people worldwide, of which approximately 5 million die and 5 million are disabled for life (Maida et al., 2020). In recent years, with the aging of the population, the risk of IS has greatly increased, resulting in great pain and economic burden to patients (Matsuzono et al., 2021). Currently, studies in this area mainly focus on the regulation of pathological mechanisms, including apoptosis, inflammation, oxidative stress, and calcium overload (Feske, 2021). Multiple genes and regulatory methods are involved in IS, such as phosphorylation signal transduction and RNA methylation modification (Zhang et al., 2020). Identifying key genes and intervening in their regulation can improve the IS prognosis and provide newer ideas for its treatment.

Recently, the role of RNA modifications in gene regulation has received increasing attention. More than 150 RNA modification methods have been discovered, of which methylation modifications are the most abundant (Chen et al., 2019). Methylation modifications include 1-methyladenosine, 5-methyluridine, 5-methylcytidine (m5C), and G methylation of m1G, m2G, and m7G, 2′-O-ribonucleoside, and N6-methyladenosine (m6A) (Yang et al., 2021). Modification of 7-methylguanosine (m7G) is one of the most common base modifications in post-transcriptional regulation. It is widely distributed in the 5′ cap region of tRNA, rRNA, and eukaryotic mRNA (Tomikawa, 2018). Zhao et al. (Zhao et al., 2021) found that m7G-regulated genes are differentially expressed and induce angiogenesis in other ischemic diseases. In addition, m7G-regulated genes play an irreplaceable role in many diseases, such as tumors and gastrointestinal diseases (Dai et al., 2021). However, the exact regulatory role of m7G-regulated genes in IS remains unclear.

To the best of our knowledge, this is the first study to explored the epigenetic role of m7G-regulated genes in IS. After screening using machine learning, we identified five m7G-regulated genes involved in IS using the middle cerebral artery occlusion (MCAO) animal model, which were clearly clustered IS patients into two m7G clusters, and the immune infiltration of each cluster was further analyzed. Through functional enrichment and the mRNA–miRNA–transcription factor (TF) network, we further revealed the biological functions and regulation modes of different m7G clusters. This study provides a novel m7G cluster method that extensively participates in the regulation of IS occurrence and treatment.

## 2 Methods

### 2.1 Data collection

Two IS-related mRNA expression profiling datasets, GSE58294 and GSE22255, were downloaded from the Gene Expression Omnibus (GEO) database using the R package “GEOquery.” GSE58294 contains 92 samples, including 23 control samples and 69 IS samples, whereas GSE22255 contains 20 patients with IS and 20 healthy individuals. These samples were all detected by GPL570 probe (Affymetrix Human Genome U133 Plus 2.0 Array). The “normalizeBetween-Arrays” function of the “limma” package was used to normalize the expression matrix. The gene probes were annotated using official symbols. We calculated the mean values if multiple gene probes matched the same gene.

### 2.2 Establishment of the middle cerebral artery occlusion (MCAO) model

In total, 200–240 g Sprague–Dawley rats were purchased from the Animal Experiment Center of Xi’an Jiaotong University. Rat MCAO model was established, as previously developed and described (Longa et al., 1989). In brief, the external carotid artery of the rat was carefully isolated and an incision was made. A suture (RWD, Shenzhen, China) with a head diameter of approximately 0.34 ± 0.01 mm was inserted from the incision in the external carotid artery into the internal carotid artery up to the middle cerebral artery. Two hours later, the suture was removed and the wound was sutured. After 3 days, the rats were euthanized. The rat brain was snap-frozen, cut into 2-mm coronal slices, and immersed in 2, 3, 5-triphenyl tetrazolium chloride (TTC) solution in a 37°C water bath for 30 min. Images were taken using a digital camera after dyeing.

### 2.3 Machine learning screens 7-methylguanosine (m7G) key genes between healthy individuals and patients with ischemic stroke (IS)

Based on previous studies on m7G, 34 m7G key regulatory genes were included in this study as study objects, including *DCP2, AGO2, CYFIP1, CYFIP2, DCPS, EIF3D, EIF4A1, EIF4E, EIF4E1B, EIF4E2, EIF4E3, EIF4G3, GEMIN5, IFIT5, LARP1, LSM1, METTL1, NCBP1, NCBP2, NCBP2L, NCBP3, NSUN2, NUDT1, NUDT10, NUDT11, NUDT16, NUDT16L1, NUDT3, NUDT4, NUDT4B, NUDT5, NUDT7, SNUPN,* and *WDR4* (Tomikawa, 2018; Chen et al., 2022). Differences in the expression patterns of these genes between patients and controls were detected using the Wilcoxon test, with a selection criterion of *p* < 0.05. Spearman correlation analysis was performed on these differentially expressed genes (DEGs), and their chromosomal locations were marked. This study utilized two widely used machine learning algorithms, random forest (RF) and support vector machine (SVM), to identify key regulators of m7G between patients with IS and controls by the “randomForest” package. The algorithm with the smaller residual was considered to be a more precise algorithm and was used. The R package “pROC” was used to calculate the area under the curve (AUC) and evaluate the accuracy of the two algorithms.

### 2.4 Quantitative real-time polymerase chain reaction

Total RNA was extracted from the ischemic penumbra of rats and from the same site in the control group using TRIzol (Invitrogen, USA). After reverse transcription, real-time PCR was performed on genes with significant m7G differences. The primer sequences for these genes are listed in Table 1. Glyceraldehyde-3-phosphate dehydrogenase (GAPDH) was used as the internal reference gene. The results are expressed as relative mRNA expression at cycle thresholds and normalized by parallel amplification of the endogenous control GAPDH. The relative mRNA expression level (target mRNA/GAPDH value) of the control group was set as 100%, and the mRNA values of the other groups were converted into fold changes after comparison with the control group.

**TABLE 1:** Specific primers used for quantitative real-time PCR.

Primer name	Sequence
GAPDH-F	TGC​CAC​TCA​GAA​GAC​TGT​GG
GAPDH-R	TTC​AGC​TCT​GGG​ATG​ACC​TT
NCBP2-F	AGC​GTG​TGG​GTT​CTG​TTT​CGT​G
NCBP2-R	CAT​ACT​GCC​TGC​CCT​CCT​TAA​AGC
CYFIP1-F	GAT​GGT​GGA​GAG​GAT​TCG​CAA​GTT​C
CYFIP1-R	CTG​GCT​AGG​GAC​TGG​TGG​ATG​G
NUDT1-F	TAC​TAC​AGC​CTC​AGC​GAG​TTC​TCC
NUDT1-R	TCC​CTC​TTA​GCC​CCA​TCC​TCA​ATG
DCPS-F	AAG​CAG​GCG​TTG​GCA​ATG​GTA​C
DCPS-R	TCC​CCA​GAG​TCC​TCA​TTC​ACC​TTC
NSUN2-F	CGC​TGC​TAT​CTG​CTC​GTC​CAT​C
NSUN2-R	CTG​TGA​GTC​TAG​GAA​TGC​TGG​ATG​C
CYFIP2-F	CCA​CCA​CCA​ACT​GAA​GGA​CAT​CAT​C
CYFIP2-R	TCT​ATG​AGG​AGG​CAG​AAC​AGG​ATG​G
EIF4E3-F	GAG​TGT​GCC​TCG​AAC​CTG​AAG​AAG
EIF4E3-R	TGG​TCG​CCT​CTC​TCC​TCT​CAT​TAA​G
EIF3D-F	CAA​CAA​GCA​GGT​CAT​CCG​AGT​CTA​C
EIF3D-R	CCT​CCT​CTT​CCT​CCT​CAT​CCT​CTT​C

### 2.5 Establishment and validation of clinical prediction models

The expression of the five m7G-related genes was packed by the “datadist” function of the “rms” package, and subsequently, the model was fitted using the “lrm” function. The “nomogram” function was used to build a suitable model and draw a nomogram by these risk genes. The total score of the nomogram was the sum of the corresponding scores assigned to each differential gene, and the score corresponded to the corresponding disease risk. The higher the score, the higher the risk of gene-induced IS development. Internal validation using the “caret” package and Bootstrap self-sampling method to derive the consistency index (C-Index). The calibration, clinical decision analysis, and receiver operating characteristic (ROC) curves were used to further evaluate the accuracy of the risk model.

### 2.6 Cluster analysis of patients with IS by m7G-regulated genes

Cluster analysis was used to distinguish different IS patient classifications based on the regulation of key m7G genes. The R package “ConsensusClusterPlus” was used to classify patients with IS into different subgroups according to experimentally validated m7G key regulatory genes. In this study, the PAM algorithm and spearman distance were used as parameters, and the sampling was repeated 1,000 times for a more stable classification. The number of clusters was determined using a cumulative distribution function. The “Rtsne” package was used to display the distribution of samples for different clusters. The expression of m7G key regulatory genes was compared between the two clusters using the Kruskal–Wallis test.

### 2.7 Predicting the immune properties of m7G key regulatory genes

The single-sample gene set enrichment analysis (ssGSEA) algorithm was used to assess the immune infiltration of samples and genes by the “gsva” package. This study analyzed 23 immune cell types using ssGSEA. These included activated B cells, activated CD4 T cells, activated CD8 T cells, activated dendritic cells, CD56 bright natural killer (NK) cells, CD56 dim NK cells, eosinophils, gamma delta T cells, immature B cells, immature dendritic cells, myeloid-derived suppressor cells (MDSCs), macrophages, mast cells, monocytes, NK T cells, NK cells, neutrophils, plasmacytoid dendritic cells, regulatory T cells, T follicular helper cells, type 1 T helper cells, type 17 T helper cells, and type 2 T helper cells. The infiltrating immune cell abundance scores in two different patient clusters were compared using the Kruskal–Wallis test. A heatmap was drawn by the “pheatmap” package to show the correlation between five m7G key regulatory genes and these immune cells and to select a key gene that best represents the cluster analysis.

### 2.8 Enrichment analysis

After cluster analysis, the DEGs between the two clusters were screened by the “limma” package, and the screening conditions were as follows: |log2 (fold change)| > 0.5, adjustment *p*-value < 0.05. Metascape (https://metascape.org/gp/index.html) is an excellent tool for pathway and biological function enrichment analysis. These genes were functionally enriched using Metascape, with output options, including Gene Ontology (GO) biological processes, canonical pathways, Kyoto Encyclopedia of Genes and Genomes pathway, and Reactome gene sets.

### 2.9 Construction of the mRNA–miRNA–transcription factor (TF) network

The STRING database (https://cn.string-db.org/) can be used to assess protein–protein interactions (PPIs). The DEGs between the two m7G clusters were inputted into the STRING database to construct a PPI network. After forming the PPI network, we performed cluster analysis on the PPI network using MCODE of Cytoscape and explored the cluster with the highest MCODE score as the key genes network. The possible binding miRNAs of the key genes were predicted using the TargetScan (https://www.targetscan.org/) and miRTarBase databases (https://www.mirbase.org/). Predicted transcription factors (TF) may bind to key genes in the Enrichr database (https://maayanlab.cloud/Enrichr/). Finally, Cytoscape 3.7.2 was used to construct the mRNA–miRNA–TF network.

### 2.10 Statistical analyses

R version 4.0.2 was applied for all statistical analyses. Between-group comparisons were made using the independent samples *t*-test and Mann–Whitney *U* test. All analyses were based on two-tailed tests, and statistical significance was set at *p* < 0.05.

## 3 Results

### 3.1 Expression patterns and differences of m7G-regulated genes in IS

**FIGURE 1:**
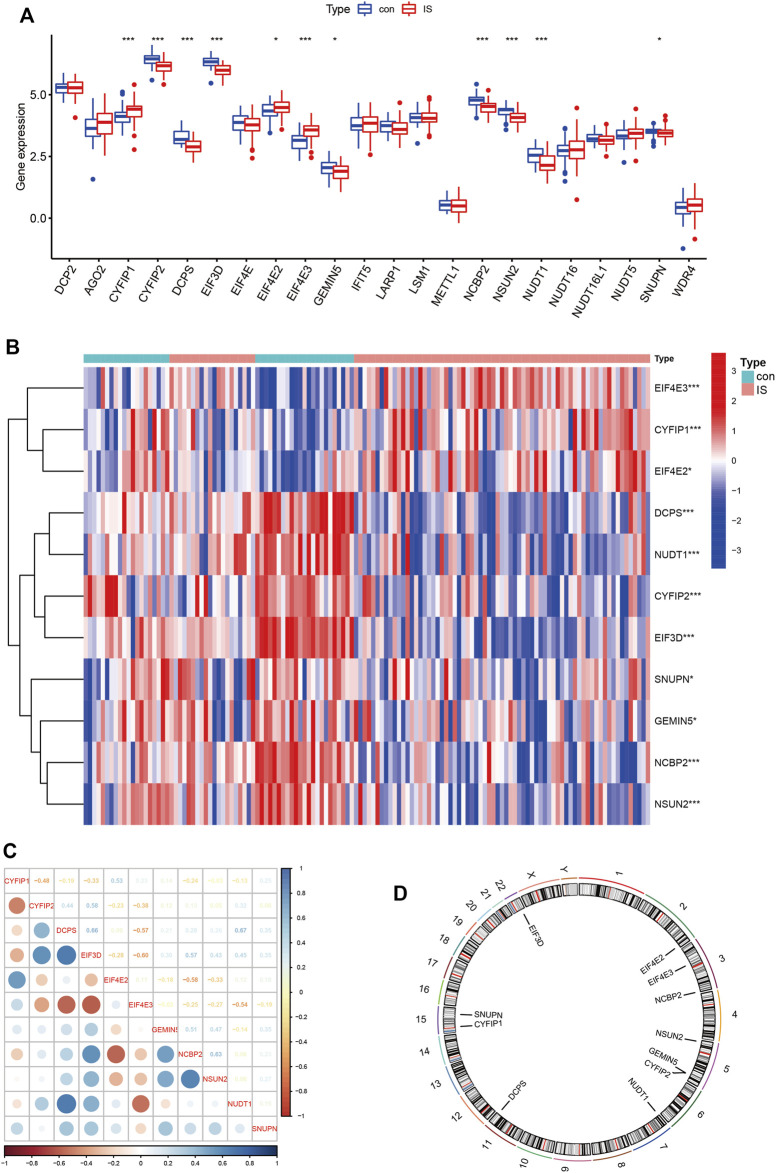
Expression patterns and differences of m7G-regulated genes in IS. **(A)** Boxplot of 34 m7G genes expression between control and IS. **(B)** Heatmap of 11 differentials expressed m7G genes between control and IS. Red represents high expression and blue represents low expression. **(C)** Correlations of m7G DEGs in IS. Blue represents positive correlation and red represents negative correlation. **(D)** Chromosomal positions of m7G DEGs. *p < 0.05, **p < 0.01, ***p < 0.001. IS: ischemia stroke; con: control; DEGs: differentially expressed genes.

We explored the differential expression of 34 m7G-regulated genes in IS and found that 11 genes were significantly differentially expressed. Among these, *CYFIP1, EIF4E2,* and *EIF4E3* were significantly upregulated in IS, whereas *CYFIP2, DCPS, EIF3D, GEMIN5, NCBP2, NSUN2, NUDT1,* and *SNUPN* were significantly downregulated (Figures 1A, B). To explore whether these m7G-regulated genes played a key role in IS, we assessed the correlation between these genes (Figure 1C). In IS, *DCPS* and *NUDT1* showed a high positive correlation (*r* = 0.67), and *EIF3D* and *EIF4E3* showed a high negative correlation (*r* = –0.60). This suggested that m7G-regulated genes play an important role in IS. We further marked the location of these genes on the chromosomes (Figure 1D).

### 3.2 Machine learning and m7G key gene screening

**FIGURE 2:**
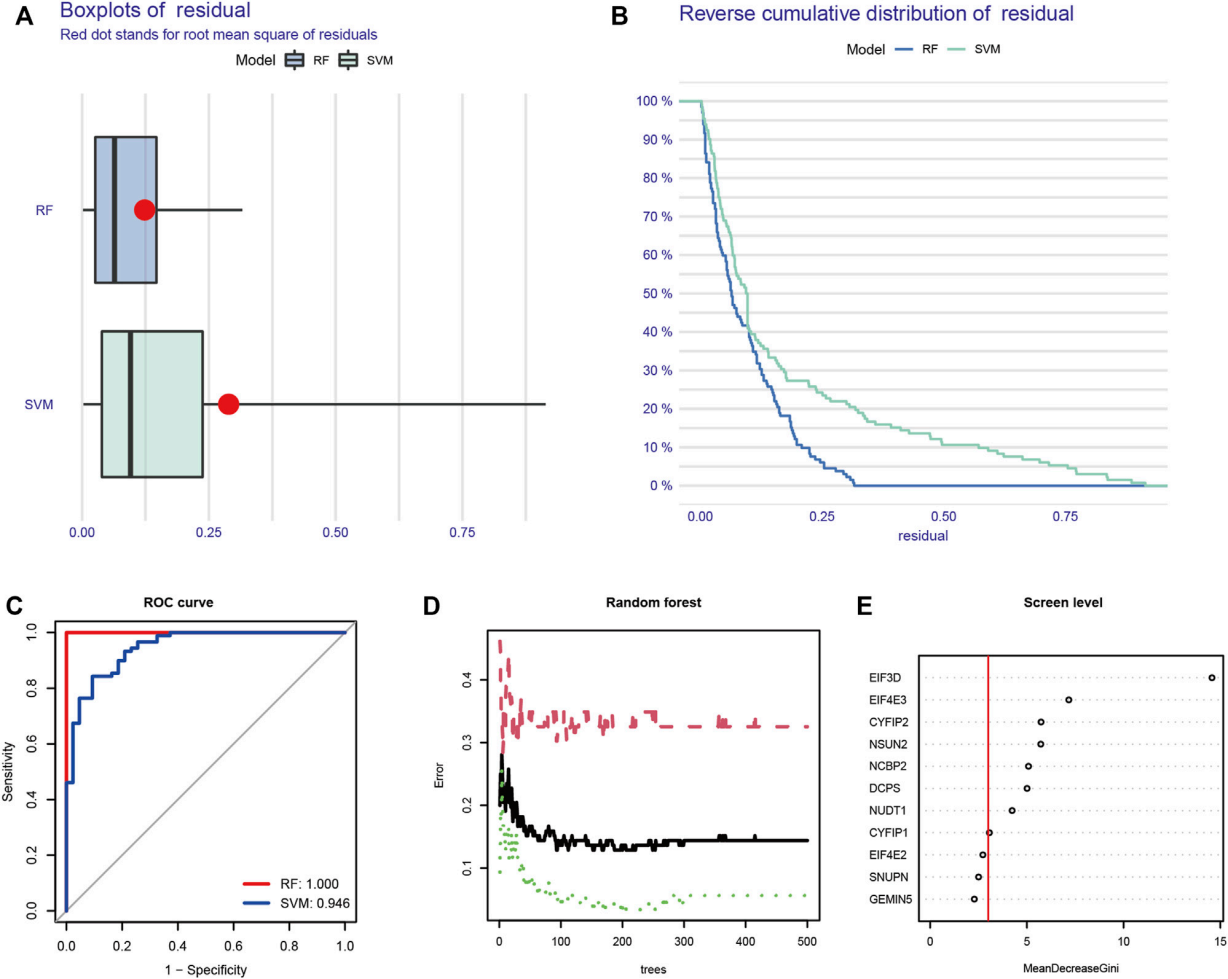
Machine learning screens m7G key regulatory genes. **(A)** Boxplot of residual in RF and SVM. **(B)** Reverse cumulative distribution of residual in RF and SVM. **(C)** ROC curve of RF and SVM. **(D)** Random forest screening of DEGs. **(E)** Screening for candidate m7G-regulated genes by RF. RF: random forest; SVM: support vector machine; ROC: receiver operating characteristic; DEGs: differentially expressed genes.

The machine learning algorithm was used to further screen for m7G key regulatory genes. We compared two machine learning algorithms and found that the residual of RF was significantly smaller than that of SVM (Figures 2A, B). In the ROC curve, the RF algorithm (AUC = 1) also showed better accuracy than SVM (Figure 2C). Therefore, the RF algorithm was selected as the machine learning algorithm in this experiment. When the number of trees was 93, the machine learning error of the RF algorithm was the smallest (Figure 2D). Finally, eight genes with an importance score greater than 3 were selected: *CYFIP1, CYFIP2, DCPS, EIF3D, EIF4E3, NCBP2, NSUN2,* and *NUDT1* (Figure 2E).

### 3.3 Expression profiles of m7G-regulated genes in the MCAO model

**FIGURE 3:**
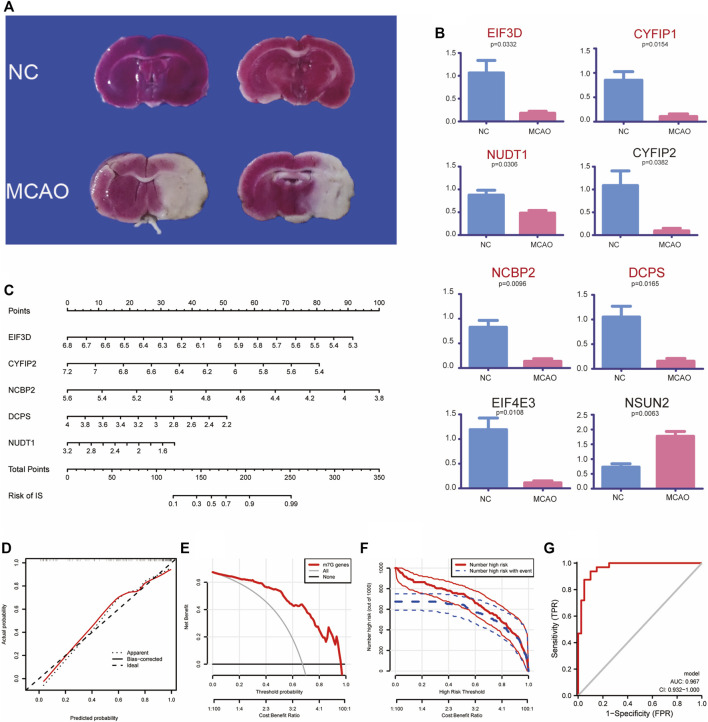
Experimental validation and clinical prediction models. **(A)** TTC verification of MCAO model. **(B)** Validation of quantitative real-time PCR analysis. **(C)** Nomogram of m7G key regulatory genes for predicting IS. Calibration curve **(D)**, Clinical decision analysis **(E,F)** and ROC curve **(G)** of nomogram. TTC: 2, 3, 5-triphenyl tetrazolium chloride; MCAO: middle cerebral artery occlusion; IS: ischemia stroke; ROC: receiver operating characteristic.

To explore the expression of m7G-regulated genes in IS, we constructed a MCAO model. We used rat brain tissue for TTC staining 3 days after modeling to verify the success of the modeling. A clear white infarct appeared in the left cerebral hemisphere of the model group, whereas the whole brain of the control group showed a red active state (Figure 3A). Eight screened m7G-regulated genes were verified using qPCR. The results showed that *EIF3D, CYFIP2, NCBP2, DCPS,* and *NUDT1* exhibited significant differences in the MCAO model, which was consistent with the differential analysis of the expression profile dataset (Figure 3B). These results confirm that these m7G-regulated genes play a significant regulatory role in IS.

### 3.4 Establishment of a clinical prediction model

We established a clinical prediction model to evaluate the risk and correlation between five key m7G-regulated genes in IS. Our nomogram showed the risk of developing IS for each gene (Figure 3C). The internal validation of the model using Bootstrap self-sampling method with 1,000 samples yielded a model C-Index of 0.888. A calibration curve was used to further confirm the accuracy of the model, which showed that the prediction model had a good accuracy (Figure 3D). The decision analysis curve also showed that m7G could predict the risk of disease more accurately (Figures 3E, F). The ROC curve (AUC = 0.967, 95% *CI* 0.932–1.000) further supported these results (Figure 3G). In conclusion, we used a clinical predictive model to accurately assess the risk of m7G-regulated genes in IS.

### 3.5 Cluster of patients with IS according to m7G key regulatory genes

**FIGURE 4:**
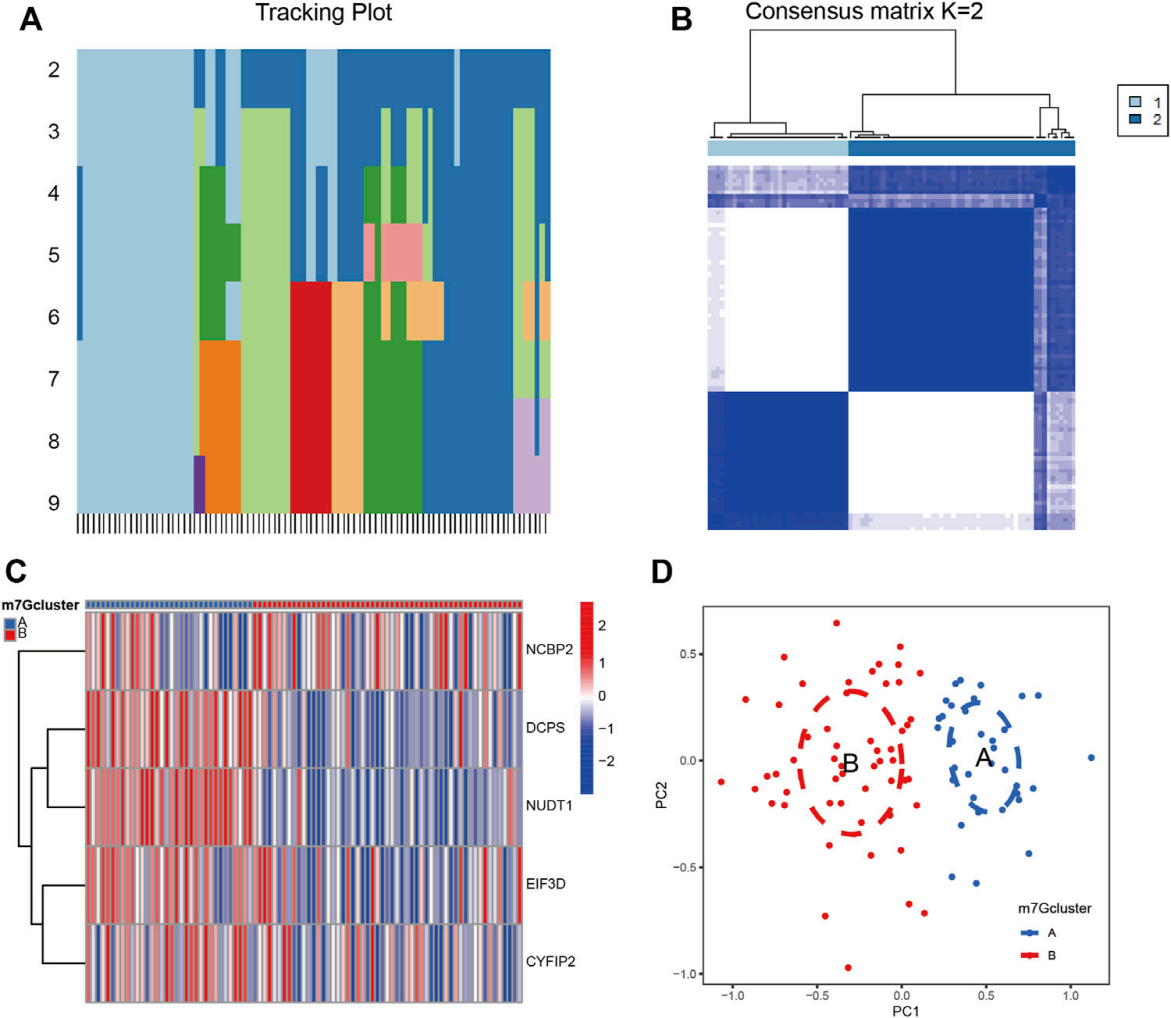
Cluster analysis of IS by m7G key regulatory genes. **(A)** Sample distribution for *k* = 2–9. **(B)** Consensus clustering matrix with *k* = 2. **(C)** Heatmap of m7G key regulatory genes between clusters. Red represents high expression and blue represents low expression. **(D)** PCA analysis between clusters. Group A and group B represent two clusters of IS patients divided according to the expression of m7G key regulatory genes. PCA: principal component analysis.

Based on the five validated key regulatory genes of m7G, we performed cluster analysis on patients with IS. The tracking plot showed that it was prudent to divide the patients into two clusters for accuracy (Figures 4A, B). We displayed the expression profiles of five m7G key regulatory genes according to these two clusters and found that their expression levels varied significantly in different clusters (Figure 4C). Principal component analysis (PCA) revealed that this clustering method could completely and accurately distinguish patients with IS (Figure 4D). Therefore, we accurately clustered patients with IS according to the expression patterns of m7G-regulated genes.

### 3.6 Immune infiltration signatures of m7G clusters

**FIGURE 5:**
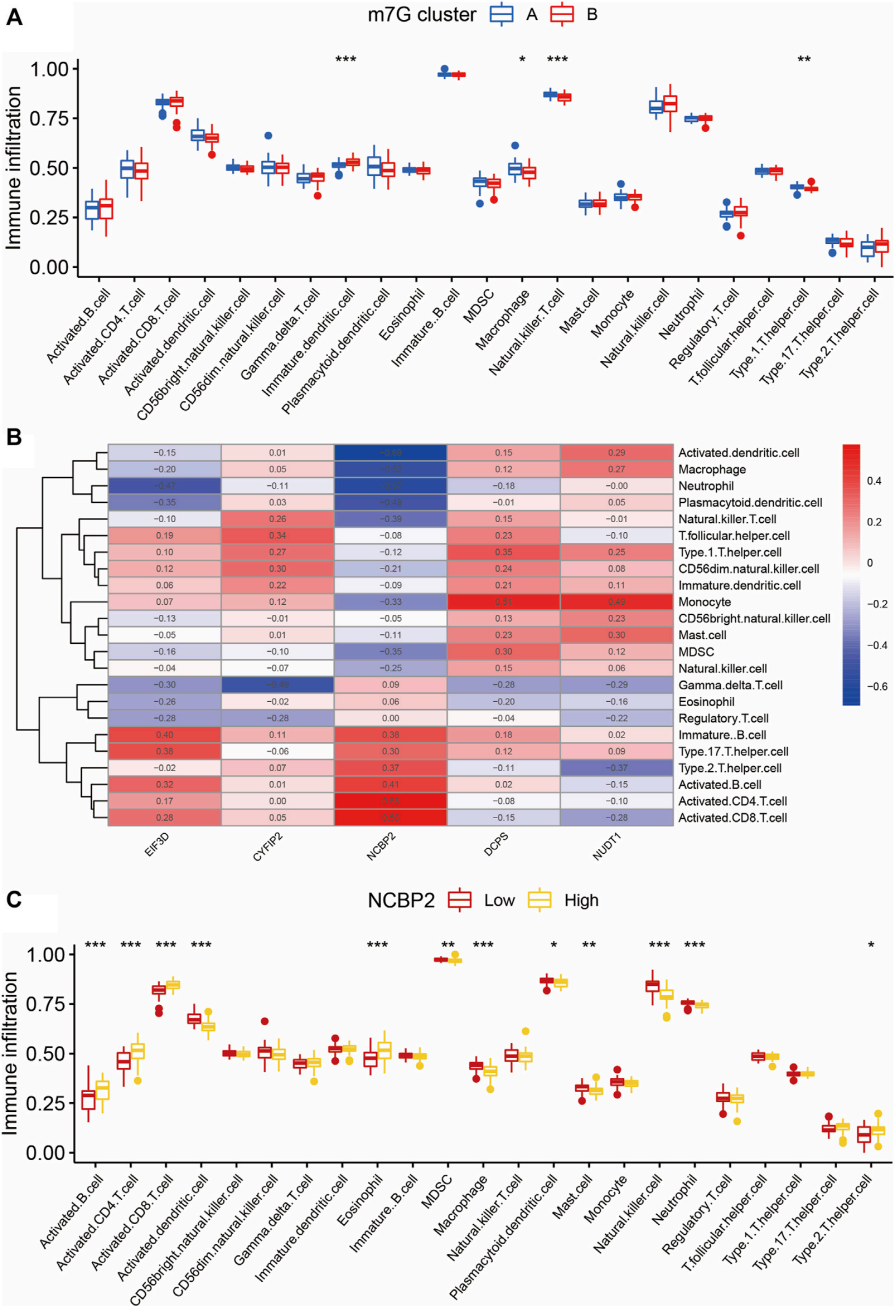
Immune infiltration analysis of two m7G clusters. **(A)** Differences in immune infiltration abundances between two m7G clusters. Group A and group B represent two clusters of IS patients divided according to the expression of m7G key regulatory genes. **(B)** Immune cell infiltration correlation heatmap of m7G key regulatory genes. Red represents positive correlation and blue represents negative correlation. **(C)** Immune infiltration analysis between clusters with different *NCBP2* expression levels. Group Low and group High represent cell clusters with low and high *NCBP2* expression, respectively. *p < 0.05, **p < 0.01, ***p < 0.001. IS: ischemia stroke.

We used the ssGSEA algorithm to evaluate the level of immune cell infiltration between different clusters to explore the differences in their immune microenvironment characteristics. We found that the four types of immune cell infiltration were significantly different between the two clusters: immature dendritic cells, macrophages, NK T cells, and type1 T helper cells (Figure 5A). The correlation of the five m7G-regulated genes experimentally identified with immune cell infiltration was also calculated (Figure 5B). Among them, the correlation of *NCBP2* was the most evident, with a maximum positive correlation coefficient of 0.58 and a maximum negative correlation coefficient of –0.69. Therefore, NCBP2 may play a critical role in immune cell infiltration. As shown in Figure 5C, among the cells with different *NCBP2* expression levels, there were more cell types with significant differences in immune infiltration, including activated B cells, activated CD4 T cells, activated CD8 T cells, activated dendritic cells, eosinophils, MDSC, macrophages, plasmacytoid dendritic cells, mast cells, NK cells, neutrophils, and type 2 T helper cells.

### 3.7 Enrichment among different m7G clusters

**FIGURE 6:**
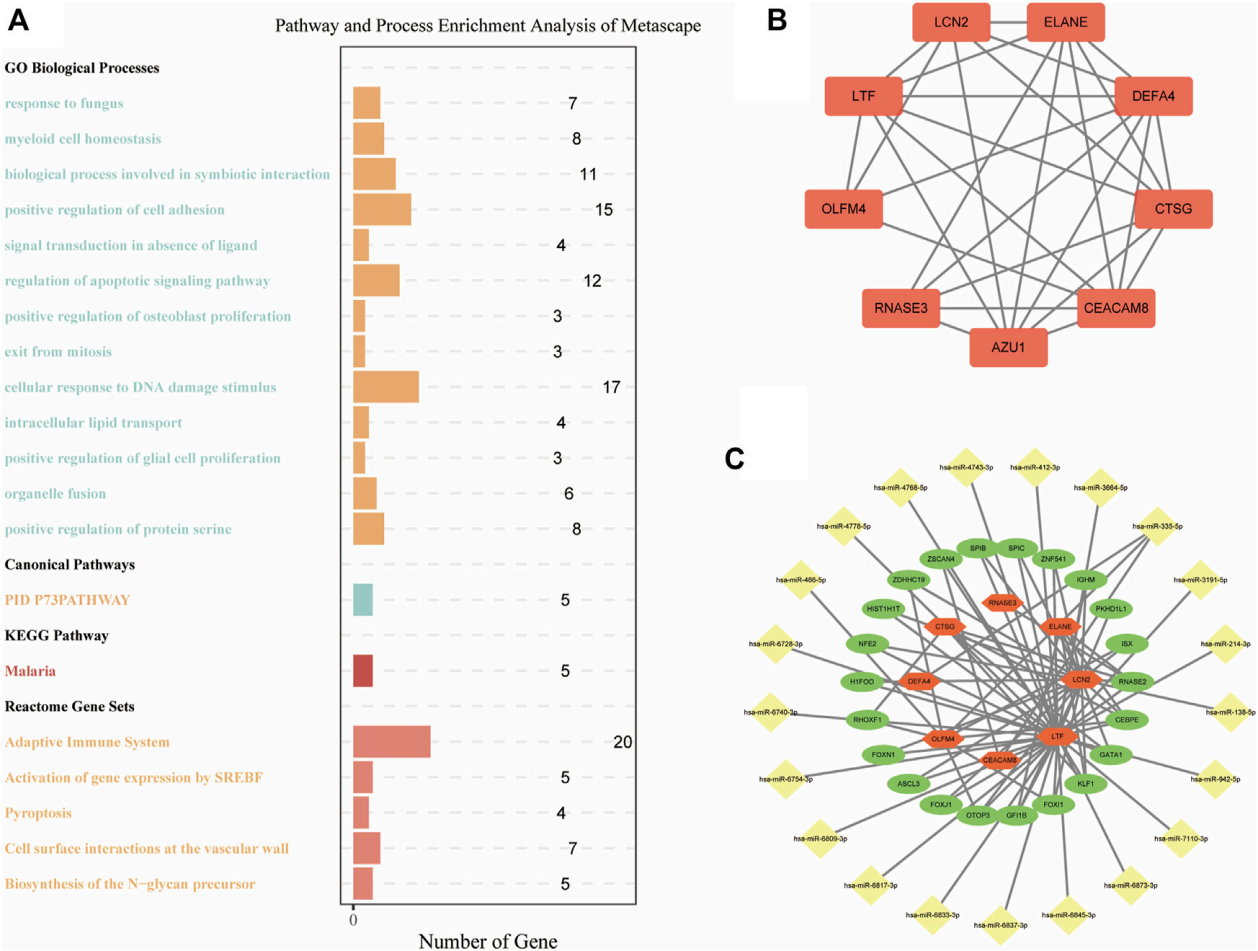
Functional enrichment and mRNA–miRNA–TF networks. **(A)** Pathway and process enrichment analysis of DEGs between m7G clusters. **(B)** PPI network of DEGs between m7G clusters. **(C)** mRNA–miRNA–TF networks of DEGs between m7G clusters. TF: transcription factor; PPI: protein–protein interactions; DEGs: differentially expressed genes.

To explore the characteristics of the biological functions under different m7G gene expression patterns, we performed biological functions and pathway enrichment analysis. Specifically, we screened 282 DEGs between the two m7G clusters (Supplementary Table S1). Metascape was used for the enrichment analysis (Figure 6A). The results showed that in the “GO Biological Processes” analysis, the “regulation of apoptotic signaling pathway” and “cellular response to DNA damage stimulus,” the mechanisms closely related to IS pathogenesis, were enriched in 12 and 17 genes, respectively. In the “Reactome Gene Set” analysis, the genes enriched in the two IS-related pathways of “adaptive immune system” and “pyroptosis” were 20 and 4 genes, respectively. This indicates that m7G-regulated genes are closely related to IS in terms of biological functions.

### 3.8 Construction of the mRNA–miRNA–TF network

DEGs between the two m7G clusters were used to build a PPI network to explore the interaction relationship between genes. There were 254 nodes and 274 edges in this PPI network (Supplementary Figure S1). After further identification of key modules and hub genes using MCODE, a PPI network with 9 nodes and 26 edges was identified, which included *LTF, LCN2, ELANE, RNASE3, CTSG, DEFA4, OLFM4,* and *CEACAM8* (Figure 6B). We combined “Targetscan” and “miRTarBase” databases to successfully predict 21 miRNAs that may bind to key regulatory genes of m7G. Next, we showed the mRNA–miRNA–TF networks (Figure 6C). Among them, *LTF* had the largest number of nodes and edges and may bind to 18 miRNAs and 19 TFs. This indicates that *LTF* plays an important regulatory role in IS.

## 4 Discussion

In this study, we discovered the epigenetic and immune microenvironmental regulatory mechanisms of m7G in IS. First, we screened out the differentially expressed m7G regulatory genes in IS. Second, we identified that *EIF3D, CYFIP2, NCBP2, DCPS,* and *NUDT1* were five key m7G-regulated genes differentially expressed in IS according to the RF algorithm and qPCR of the MCAO model. The risk impact of these genes on developing IS was assessed separately, and patients with IS were divided into two clusters based on these genes. Finally, immune infiltration between the two clusters and the functional enrichment and regulatory network of differential genes were also revealed.

IS is a complex disease that involves multiple molecular mechanisms and methylation modifications. Chokkalla et al. (Chokkalla et al., 2019) found that regulation of m6A methylation is involved in IS development and can be considered an important marker of IS. Zhang et al. (Zhang et al., 2020) found that YTH domain-containing 1 acts as an m6A reader and alleviates IS by promoting the activation of the AKT signaling pathway. However, studies on m7G and IS are limited. Therefore, our study provides evidence for epigenetic studies on methylation and IS.

We screened key m7G-regulated genes in IS and further verified this using the rat MCAO model by qPCR, which greatly improved the accuracy of the screening. These genes included *EIF3D, CYFIP2, NCBP2, DCPS,* and *NUDT1*. *EIF3D* and *NUDT1* have been shown to play important regulatory roles in tumor and immune infiltration (Huang et al., 2019; Huang et al., 2022). *CYFIP2* has been shown to play vital regulatory role in the central nervous system (Schaks et al., 2020). *NCBP2* and *DCPS* are believed to be involved in neurogenesis, which may inextricably be associated with IS (Singh et al., 2020; Salamon et al., 2022). However, the specific mode of regulation between them and the IS has not yet been studied. Therefore, our study successfully confirmed their close correlation with IS using an animal model.

Machine learning and clinical predictions are excellent tools for bioinformatics analysis, enabling accurate assessment of disease regulatory mechanisms and risks. The algorithm we used, RF, has been used for long-term outcome prediction of mortality and morbidity in patients with stroke. Heo et al. (Heo et al., 2019) found that the RF algorithm can also predict the long-term prognosis of IS. Our study not only selected the key genes with the RF algorithm but also proved that RF was more suitable for our study, which provides evidence for the precise selection of the appropriate machine learning. Nomograms have been widely used in clinical prediction models of stroke. Yuan et al. (Yuan et al., 2020) used a nomogram to accurately predict the risk of stroke using multiple risk factors, including hypertension, diabetes, and smoking. Our study further refines the risk factors for genes, providing a more precise theoretical basis for the prevention and treatment of stroke through molecular mechanisms.

Our study makes the first attempt to cluster patients with IS into two defined clusters based on m7G key gene expression profiles, as well as presents novel methodologies for identifying different types of patients with IS and their precise treatment. In addition, we analyzed the differences in immune infiltration between the two clusters. Li et al. (Li et al., 2022) clustered patients with liver cancer by m7G-regulated gene expression patterns. In our study, the immune infiltrating cells with significant differences between the different clusters were immature dendritic cells, macrophages, NK T cells, and type 1 T helper cells. Therefore, we confirmed that m7G-regulated genes have profound effects on immune cell infiltration and play different immune regulatory roles in various diseases. We also found that these differences in immune cell infiltration were closely related to *NCBP2*.

Differential m7G gene expression profiles between clusters were screened and functionally enriched and mRNA–miRNA–TF networks were established. Several reports have suggested that *LCN2* can mediate the phagocytosis of astrocytes to trigger demyelination, which exacerbates IS (Wan et al., 2022). The critical role of the miR-214–*LTF*–*FOXJ1* axis was also observed in our study. Although *LTF* is believed to mediate neuronal ferroptosis in hemorrhagic stroke, it has rarely been reported in IS (Zhao et al., 2018). MIR-214 attenuates neuronal apoptosis and ferroptosis in IS, and *FOXJ1* is believed to induce neurogenesis (Devaraju et al., 2013; Lu et al., 2020). Therefore, we hypothesized that the miR-214–*LTF*–*FOXJ1* axis may play an important regulatory role in IS, thus becoming an important molecular target for the prevention and treatment of IS. However, the specific role of this axis has not yet been verified, which may become the focus of our next study.

This study has its own limitations. First, only vivo experiments but no vitro cell experiments were performed. This may be improved in subsequent studies. Second, although we innovatively discovered the miR-214–*LTF*–*FOXJ1* axis, this could not be verified by basic experiments. Third, although we were able to establish detailed predictions on the mediation network of m7G, we did not further explore therapeutic drugs based on this, which is insufficient for clinical guidance. In addition, we still need to obtain more clinical data from patients as an analysis basis to augment the accuracy of assessment and prediction.

## Data Availability

The datasets presented in this study can be found in online repositories. The names of the repository/repositories and accession number(s) can be found in the article/Supplementary Material.
