# Supplementary figures and images for: Experimental verification and comprehensive analysis of m7G methylation regulators in the subcluster classification of ischemic stroke
Source: Front Genet. 2023 Jan 4;13:1036345. doi: 10.3389/fgene.2022.1036345 (PMC9845407; doi:10.3389/fgene.2022.1036345)

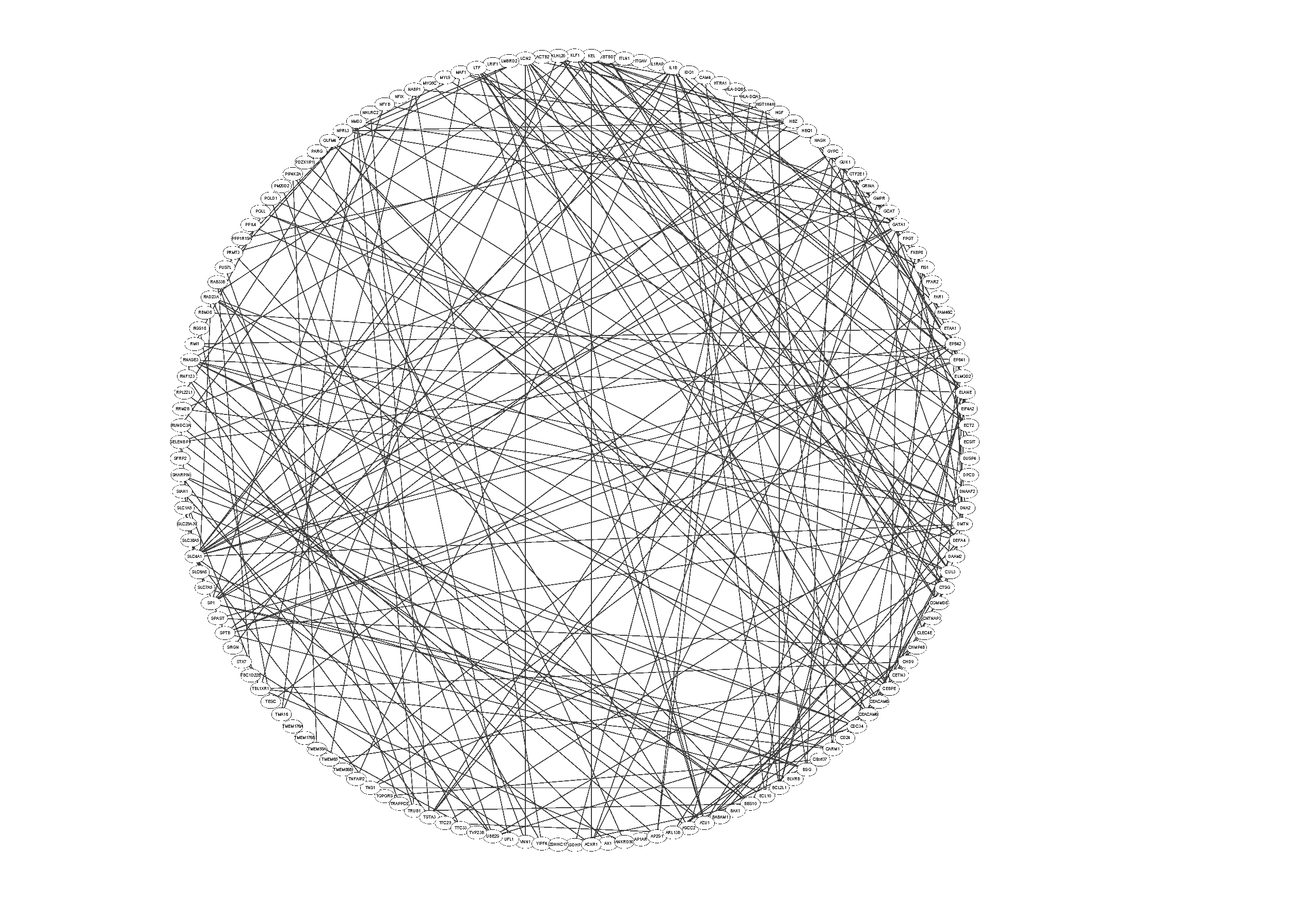

Supplement: Supplementary file 1 [file Image1.TIF]
